# A Japanese translation of the Swedish Universities Scales of Personality

**DOI:** 10.48101/ujms.v129.10349

**Published:** 2024-03-21

**Authors:** Lykke Silfwerbrand, Lisa Ekselius, Yasuharu Koike, Malin Gingnell

**Affiliations:** a Department of Medical Sciences, Uppsala University, Uppsala, Sweden; b Institute of Innovative Research, Tokyo Institute of Technology, Yokohama, Japan; c Department of Women’s and Children’s Health, Uppsala University, Uppsala, Sweden; d Department of Psychology, Uppsala University, Uppsala, Sweden

**Keywords:** Swedish Universities Scales of Personality (SSP), cross-cultural translation, reliability, Swedish to Japanese, personality inventory

## Abstract

**Background:**

The Swedish Universities Scales of Personality (SSP) is a personality measurement tool with a short test battery of high psychometric quality, previously not availiable in Japanese.

**Methods:**

We translated the SSP into Japanese and administered it to 103 Japanese nationals. For 11 of the 13 SSP scales in the Japanese version of the SSP (SSP-J11), the Cronbach’s alpha ranged from 0.50 to 0.82 with good internal scale reliability.

**Results:**

A principal factor analysis replicated the previous work by identifying the same three principal dimensions of Neuroticism, Aggression, and Extraversion factors.

**Conclusion:**

The resulting three-factor SSP-J11 shows acceptable reliability and should provide informative insights about personality traits in research and clinical practice in a Japanese context.

## Introduction

Personality assessment is crucial for the study of social interactions. Short test batteries with high psychometric quality which are based on traits thought to have neurobiological correlates are few but existing (1). Also, such reliable personality assessment instruments that are cross-culturally translated for international use are scarce (1, 2).

Personality can be described as the most important features in a person’s social and emotional functioning, distinguishing behaviors and patterns of actions (3). The differences in personalities can be attributed to individual traits. Personality traits are relatively stable, consistent, and enduring individual characteristics that can be disentangled by analysis of behaviors, attitudes, feelings, and habits (4). Personality traits can be measured by specialized tools, including self-reports, structured observations, or data collected from individuals close to the target person. Methods for data collection could be checklists, inventories, or opinion surveys (5). A widely used personality inventory, the five-factor model of personality, primarily uses lexical analysis to define traits (6). Other personality assessment inventories, such as Eysenck’s and Sjöbring’s models, have been based on trait dimensions with biological correlates and implications for information processing and psychopathology (7, 8).

The Swedish Universities Scales of Personality (SSP), commonly interpreted as a three-dimensional personality trait assessment inventory, can be used for clinical and non-clinical populations (9). Currently, the SSP is available in several languages, including English, Chinese, and Estonian (7–9), but not yet in Japanese. The construction of the SSP forerunner, the Karolinska Scales of Personality Inventory (KSP), used concepts defined in Eysenck’s and Sjöbring’s models and was developed to assess the vulnerabilities of psychopathology (10–12). The SSP is a shortened and psychometrically enhanced version of the KSP. It is a short personality assessment instrument that uses timeless and straightforward language suitable for healthy people and psychiatric patients. There is an insignificant difference in results between female and male respondents. The test battery is a pool of scales from which a smaller number of scales can be extracted for research objectives. It can also serve as a comprehensive assessment tool for several personality traits (9). However, the SSP is often interpreted with three factors: general neuroticism, aggression, and extraversion measures. Because the SSP is a short test and psychometrically of high quality, it can serve as a valuable tool for various purposes and situations in clinical and research work.

This study has two principal aims: 1) to translate SSP into Japanese with the meaning of each item preserved concerning the Japanese language and culture and 2) to evaluate the reliability of the Japanese translation of the SSP.

## Materials and methods

### Swedish Universities Scales of Personality

SSP consists of 91 items (seven for each of the 13 scales) describing various ways of behavior. The subscales reflect the following dimensions of personality: Somatic Trait anxiety (STA), Psychic Trait Anxiety (PsTA), Stress Susceptibility (SS), Lack of Assertiveness (LA), Embitterment (E), Trait Irritability (TI), Mistrust (M), Verbal Trait Aggression (VTA), Physical Trait Aggression (PhTA), Social Desirability (SD), Impulsiveness (I), Adventure Seeking (AS), and Detachment (D). For scale descriptions, see Table 1. For each of the 91 items, there are four possible responses: 1 = ‘Does not apply at all’, 2 = ‘Does not apply very well’, 3 = ‘Applies pretty much’, and 4 = ‘Applies completely’. Each reply is scored one, two, three, or four points.

**Table 1:** Descriptions of scale traits.

Scale name	Abbreviation	For persons with high scores in the respective scales, the personality traits can be described as follows
Somatic trait anxiety	STA	A tense and restless personality with autonomic disturbances
Psychic trait anxiety	PsTA	A worrying and anticipating personality that lacks self-confidence
Stress susceptibility	SS	Feeling of uneasiness when urged to speed up and easily fatigued
Lack of assertiveness	LA	A personality that cannot speak up and be self-assertive in social situations
Impulsiveness	I	A personality that acts impulsively, without planning
Adventure seeking	AS	A personality that avoids routine and needs action
Detachment	D	A ‘schizoid’ personality that is withdrawn and avoids involvement with others
Social desirability	SD	Personality traits that are socially conforming, friendly, and helpful
Embitterment	E	An unsatisfied personality that blames and envies others
Trait irritability	TI	An irritable personality that lacks patience
Mistrust	M	A suspicious personality that distrusts other people’s motives
Verbal trait aggression	VTA	A personality that gets into arguments and beats people when annoyed
Physical trait aggression	PhTA	A personality of getting into fights, starting fights, and hitting back

*Note*. Scale descriptions from the Swedish version of the SSP (7).

### Translation method

The SSP was translated from Swedish into Japanese (hereafter referred to as the SSP-J11) and then back-translated to Swedish. The translation process followed the specifications provided by RAND Health Care (13). There were five translators (four Japanese and one Swedish). All four Japanese translators have lived in Sweden for at least 5 years and speak fluent Swedish. All translators held academic degrees. The research group was continually discussing changes and adaptations during the process. The primary focus was on reliably representing each item’s initial psychometric meaning and face value. Items in the SSP-J11 were kept in the same order as in the Swedish version.

### Participants and power

Some 103 persons took part, 62 (60%) males and 41 (40%) females (Table 2). Participants were recruited from two sources: 1) a group of 47 Japanese nationals, which were adult employees, also taking part in another study at a large technical university and 2) from a convenience sample of 56 Japanese technology university students. The questions were answered in privacy over the Internet, and all participants provided written consent before taking part in the study.

**Table 2:** Participant data for the 103 participants who answered the questionnaire, 40% of the participants were women.

Years of age	All	Women	Men
18–29	29	8	21
30–39	6	1	5
40–49	14	4	10
50–59	41	20	21
60–69	11	7	4
>70	2	1	1
Total	103	41	62

An 80% power for the study was estimated using a two-tailed independent one-sample t-test. An expected effect size of 0.3 with an error probability of 5% would require 90 participants. To compensate for a potential data loss of approximately 10%, oversampling was considered with 103 participants.

### Ethics

This study received ethical approval from the Tokyo Institute of Technology, Japan: Validation study of SSP into Japanese (dnr 2018174). For analysis of the data in Sweden, this study received ethical approval from the Swedish Ethical Review Authority (dnr 2021-05482-01).

### Statistical methods

For direct comparison to the original SSP publication, the same statistical methods were applied. The data were processed using modules and programs in Python on Jupyter (14). To compare the results with the original data, means and standard deviations were calculated for each scale for the whole sample, and women and men separately. T-tests between female and male responses were calculated scalewise for all variables. The Cronbach’s alpha for the scales was deduced for the whole group and women and men separately to assess internal reliability. Inter-scale correlations were calculated to determine how closely the scales were related. Kaiser–Meier–Olkin (KMO) measurement was applied to test that the proportion of variance of the items was adequate for factor analysis. Finally, factor analysis was derived using principal component analysis with varimax rotation to mirror the analysis used for SSP originally (9). Using the same setup allowed to assess whether this analysis yielded similar factor loadings as the original data. For power calculations, G Power software was used (15).

## Results

Descriptive statistics for the 13 scales are presented in Table 3. Mean scale results were on average 2.44, with a standard deviation of 0.519. There were no significant differences in the t-tests between the scores of women and men on any of the scales. (Data not shown.)

**Table 3:** SSP-J11 scale means and standard deviations and gender differences.

Scale name	Tot		Women		Men		SSP Tot (7)	
	*M*	(*SD*)	*M*	(*SD*)	*M*	(*SD*)	*M*	(*SD*)
Somatic trait anxiety	2.11	(0.62)	2.19	(0.56)	2.05	(0.66)	1.89	(0.58)
Psychic trait anxiety	2.48	(0.62)	2.45	(0.55)	2.50	(0.67)	2.02	(0.61)
Stress susceptibility	2.53	(0.34)	2.59	(0.33)	2.50	(0.34)	2.03	(0.49)
Lack of assertiveness	2.43	(0.48)	2.48	(0.46)	2.40	(0.50)	2.09	(0.57)
Impulsiveness	2.48	(0.52)	2.54	(0.22)	2.44	(0.58)	2.25	(0.52)
Adventure seeking	2.76	(0.56)	2.72	(0.51)	2.78	(0.60)	2.46	(0.59)
Detachment	2.51	(0.36)	2.59	(0.29)	2.47	(0.40)	2.09	(0.53)
Social desirability	2.86	(0.50)	2.84	(0.49)	2.88	(0.51)	2.87	(0.40)
Embitterment	2.45	(0.44)	2.48	(0.32)	2.43	(0.51)	1.80	(0.50)
Trait irritability	2.30	(0.54)	2.29	(0.59)	2.31	(0.51)	2.35	(0.56)
Mistrust	2.44	(0.60)	2.45	(0.64)	2.43	(0.58)	1.98	(0.53)
Verbal trait aggression	2.31	(0.49)	2.30	(0.41)	2.32	(0.54)	2.17	(0.54)
Physical trait aggression	2.11	(0.68)	2.05	(0.65)	2.15	(0.71)	2.05	(0.65)

SSP: Swedish Universities Scales of Personality; SD: standard deviation.

### Internal reliability

All but two scales had a Cronbach’s alpha value of ≥0.5 (range 0.56–0.84) for the whole sample (Table 4). SS had a Cronbach’s alpha value of 0.0091 for the whole group (women: 0.14 and men: −0.10) and D 0.18 for the whole group (women: −0.27 and men: 0.30). E showed a borderline value of 0.50 (women: 0.11 and men: 0.61). Slightly higher values were found in LA with 0.54 (women: 0.51 and men: 0.57) and VTA with 0.62 (women: 0.43 and men: 0.70). These three and the eight scales in which the Cronbach’s alpha was ≥0.70 were retained for the in-depth analyses, whereas the SS and D scales were excluded due to low internal consistency*.*

**Table 4:** Cronbach’s α of the Japanese and Swedish versions of the SSP.

Scale name	Cronbach’s α (95% Cl)	Cronbach’s α women (95% Cl)	Cronbach’s α men (95% Cl)	Cronbach’s α Swedish version of the SSP (7)	Mean inter-item correlation of the SSP (7)
Somatic trait anxiety	0.75 (0.67, 0.82)	0.69 (0.52, 0.82)	0.79 (0.70, 0.86)	0.75	0.31
Psychic trait anxiety	0.82 (0.76, 0.87)	0.78 (0.67, 0.87)	0.84 (0.77, 0.90)	0.82	0.40
Stress susceptibility	0.058 (−0.25, 0.31)	0.13 (−0.34, 0.49)	−0.0049 (−0.44, 0.34)	0.74	0.28
Lack of assertiveness	0.54 (0.40, 0.67)	0.51 (0.24, 0.71)	0.57 (0.38, 0.71)	0.78	0.33
Impulsiveness	0.70 (0.61, 0.78)	0.57 (0.33, 0.74)	0.75 (0.65, 0.84)	0.73	0.28
Adventure seeking	0.75 (0.67, 0.82)	0.68 (0.51, 0.81)	0.79 (0.70, 0.86)	0.84	0.42
Detachment	0.15 (−0.13, 0.38)	−0.22 (−0.34, 0.49)	0.27 (−0.05, 0.52)	0.77	0.32
Social desirability	0.70 (0.60, 0.78)	0.68 (0.51, 0.81)	0.70 (0.58, 0.80)	0.59	0.17
Embitterment	0.50 (0.33, 0.63)	0.071 (−0.44, 0.45)	0.61 (0.44, 0.74)	0.75	0.30
Trait irritability	0.70 (0.60, 0.78)	0.78 (0.66, 0.87)	0.64 (0.48, 0.76)	0.78	0.34
Mistrust	0.81 (0.75, 0.86)	0.85 (0.76, 0.91)	0.79 (0.70, 0.86)	0.78	0.34
Verbal trait aggression	0.62 (0.50, 0.72)	0.43 (0.11, 0.66)	0.70 (0.57, 0.80)	0.74	0.29
Physical trait aggression	0.84 (0.79, 0.86)	0.82 (0.72, 0.89)	0.86 (0.79, 0.90)	0.84	0.43

SSP: Swedish Universities Scales of Personality; CI: confidence interval.

### Inter-scale correlation

Two inter-scale correlations in the SSP-J11 scales are equal to or slightly above 0.6 (Table 5). These include the relationship between the two anxiety-prone scales (STA and PsTA) and the correlation between STA and E. More than 95% of the correlations were <0.50, with 34% < 0.40. The absolute median inter-scale correlation was 0.44. Spearman correlation analysis rendered very similar results, see Appendix A.

**Table 5:** Correlation matrix for the SSP-J11 scales.

	STA	PsTA	LA	I	AS	SD	E	TI	M	VTA	PhTA
STA	–										
PsTA	0.60***	–									
LA	0.38***	0.58***	–								
I	0.10	−0.11	−0.060	–							
AS	−0.010	−0.050	−0.10	0.43***	–						
SD	−0.28**	−0.19	−0.16	0.087	0.38***	–					
E	0.61***	0.59***	0.37***	0.13	0.12	−0.24*	–				
TI	0.53***	0.39***	0.31**	0.086	−0.046	−0.45***	0.45***	–			
M	0.49***	0.48***	0.39***	−0.091	0.061	−0.27**	0.47***	0.50***	–		
VTA	0.26**	−0.068	−0.17	0.028	−0.11	−0.30**	0.26**	0.51***	0.20*	–	
PhTA	0.27**	0.12	−0.046	0.076	0.042	−0.28**	0.37***	0.47***	0.28**	0.59***	–

STA: Somatic trait anxiety; PsTA: Psychic trait anxiety; LA: Lack of assertiveness; I: Impulsiveness; AS: Adventure seeking; SD: Social desirability; E: Embitterment; TI: Trait irritability; M: Mistrust; VTA: Verbal trait aggression; PhTA: Physical trait aggression.

* *p* < 0.01,

** *p* < 0.001,

*** *p* < 0.0001.

### Factor analysis

Factor analysis for correlations of the scales in the SSP-J11 was calculated using principal component analysis and rotated to simple structure using the Varimax method to identify factors with eigenvalues >1 (Table 6). The KMO measurement was 0.76. The first factor corresponded to the STA, PsTA, LA, E, and M scales, and the second factor included SD with a negative loading, TI, VTA, and PhTA. Two scales, I and AS, loaded on the third factor. Thus, the first factor assesses traits of neuroticism, the second various forms of aggression, and the third broadly extraversion factors. The cumulative explained variance was 66%.

**Table 6:** Principal factor analysis with varimax rotation of the 11 scales in the SSP-J11.

Scale Name	Abbrevations	Factor 1	Factor 2	Factor 3
Somatic trait anxiety	STA	**0.74**	0.33	0.066
Psychic trait anxiety	PsTA	**0.87**	−0.053	−0.091
Lack of assertiveness	LA	**0.77**	−0.23	−0.16
Impulsiveness	I	−0.0070	0.13	**0.75**
Adventure seeking	AS	0.037	−0.082	**0.87**
Social desirability	SD	−0.24	**−0.51**	0.48
Embitterment	E	**0.72**	0.33	0.20
Trait irritability	TI	0.52	**0.64**	−0.040
Mistrust	M	**0.67**	0.27	−0.030
Verbal trait aggression	VTA	−0.049	**0.88**	−0.036
Physical trait aggression	PhTA	0.12	**0.80**	0.11
Eigenvalue		3.9	1.8	1.6

*Note*. The bottom row shows the eigenvalues > 1 of the factors.

Bold scale values indicate which factor the scale is loading on.

## Discussion

Our results show that a translated and reduced version of the SSP, ‘SSP-J11’, may be reliable to use in a Japanese context. The internal reliability of the two subscales SS and D was however not acceptable.

In line with the original SSP, the SSP-J11 was interpreted as a three-factor model solution, which is constructed based on psychopathological and psychobiological theories (9, 10, 16, 17). The common scales of the SSP and the SSP-J11 have very similar loadings. The neurobiological underpinnings of personality traits are an area of active research. Since the establishment of the Swedish version of the SSP and its predecessors, some individual traits have been possible to link to neurobiology. Trait neuroticism has been linked to low serotonin uptake activity (18, 19), and low platelet monoamine oxidase activity has been associated with traits, such as extraversion (12, 20, 21). Aggression traits have been indicated to have genetic sources and relate to low activation of the MAOA gene (22, 23). However, more research is needed, and the translated version of the SSP into the SSP-J11 now allows for this possibility in a Japanese context.

The Cronbach’s alphas for the 11 scales in the SSP-J11 have acceptable values (range 0.50 to 0.82), which are similar to the Swedish version (range 0.59 to 0.84). Eight out of the 11 scales have alpha values **≥**0.7. Three more with values of 0.5–07 were also deemed acceptable since there is a small number of items in the scales (24). 95% of the inter-scale correlations are <0.50, and median intercorrelations are 0.44, indicating moderate correlation and acceptable reliability (25). Based on previous research using other scales, trait anxieties are likely rated higher by women and trait aggression higher by men (26, 27), but the present study could not detect such gender differences. Based on the power of this study, we cannot draw any firm conclusion about gender differences but we note that our results in this aspect are similar to those reported for the Swedish version of the SSP, that is, no observed gender differences. The SSPJ-11 has a middling KMO measurement of its proportion of variance, making factor analysis relevant. Taken together, the replication of the three-factor solution together with the low inter-scale correlation indicates that the SSP-J11 scales are reliable also in the Japanese context.

Introducing a new cross-culturally reliable personality assessment instrument offers unique research and clinical work opportunities, but generalizing personality tests across cultures can also be challenging. Translations need to sustain the conceptual meaning and psychometric equivalence for items in different cultural settings (2, 28). The low Cronbach’s alpha values for the scales SS and D may derive from such translation and cultural difficulties. Despite our efforts to translate the initial meaning of every item and respect cultural differences, the items of these two scales may have been interpreted differently in Japan and Sweden. In these two scales, the short questions may describe the situation too vaguely for the Japanese context, making them difficult to interpret. For example, Gustavsson et al. (9) depicted persons with high SS scores as ‘Easily fatigued, feeling uneasy when urged to speed up’. The scale includes such items as ‘I easily feel pressure when told to speed up my work’. (仕事のスピードを上げるように 言われるとすぐストレスを感じてしまう。/ Jag blir lätt stressad när jag uppmanas att skynda på med ett arbete.) and ‘I think I have less energy than most people I know’. (ほとんどの 知りあいに比べて，自分は活力が少ない方だと思う。/ Jag tycker att jag orkar mindre än de flesta i min bekantskapskrets.). Stress level has been reported as higher and more diverse in the Japanese context, which can ultimately influence the level of additional stress one can sustain^1^ (29). The world value survey also notes that the freedom of choice and control is estimated higher among Swedish people than Japanese World Values Survey Association, 2021 (30). Taken together, the items related to the pressure to sustain stress may need to be assessed differently in the two cultures. Similarly, the low reliability for D could be due to different cultural contexts and too vaguely posed questions.^2^ Gustavsson et al. (9) described personalities with high D scores as ‘Avoiding involvement in others, withdrawn’. D includes items such as ‘I feel uncomfortable when people take me into their confidence’ (人に心を許されると居 心地悪く感じる。/ Jag känner mig besvärad då folk kommer till mig med personliga förtroenden.) and ‘I prefer not to get involved in other people’s problems’. (できれば，他人の問題 に首をつっこみたくない。/ Jag föredrar att slippa engagera mig i andra människors problem.) Describing oneself on a scale of D may be more uncomfortable in the Japanese than in the Swedish context, especially as the expected personal distance is generally reported larger in Japan than in western cultures (29–32). Hence, in analogy with the discussion about SS, the statements in SSP may not be sufficiently fine-tuned to capture the essence of D within the Japanese context. However, the power of the current study does not allow for detailed analyses of isolated items. Future studies with larger sample sizes are warranted to elucidate the mechanisms for the low reliability of D and SS.

A limitation of the study is that the participants (employed, mostly middle-aged, adults, and students of technology) may not represent the general Japanese population. Also, although the study has at least 80% power for a small to medium effect size with a 5% error probability, it could be on the small side for some effects, such as gender differences. In addition, the sample size does not allow for analyses on the level of individual items in accordance to the COSMIN risk of bias checklist (33, 34), but this translation of SSP should anyway be relevant on scale and factor levels.

Based on this study, the reliability of the SSP-J11 seems acceptable on the level of scales and factors and may be used for clinical work and research in a Japanese setting. The SSPJ-11 inventory in Japanese can be supplied from the corresponding author.

## Data accessibility

The translation of the SSP into Japanese, SSPJ-11, is available for academic purposes from the corresponding author. The analysis code for this study can be supplied from the corresponding author, but the materials are not available. This study was not preregistered.

## Footnotes

1 Personal dialogue with professional translator of Japanese to Swedish, Yukiko Duke, in February 2022.

2 Personal dialogue with professional translator of Japanese to Swedish, Yukiko Duke, in February 2022.

## Appendix A

**Table 1:** Spearman’s correlation analysis of the SSP-J11 scales.

	STA	PsTA	LA	I	AS	SD	E	TI	M	VTA	PhTA
STA	–										
PsTA	0.62***	–									
LA	0.42***	0.54***	–								
I	0.12	−0.09	−0.010	–							
AS	−0.0028	0.0014	−0.10	0.36***	–						
SD	−0.24*	−0.19*	−0.24*	0.053	0.33***	–					
E	0.63***	0.59***	0.35***	0.13	0.15	−0.27**	–				
TI	0.48***	0.38***	0.32***	0.13	−0.052	−0.44***	0.46***	–			
M	0.49***	0.45***	0.38***	−0.11	0.033	−0.24*	0.45***	0.45***	–		
VTA	0.20*	−0.048	−0.10	0.068	−0.13	−0.28**	0.25**	0.50***	0.20*	–	
PhTA	0.22*	0.13	0.021	0.0036	0.024	−0.22*	0.36***	0.42***	0.30**	0.53***	–

STA: Somatic trait anxiety; PsTA: Psychic trait anxiety; LA: Lack of assertiveness; I: Impulsiveness; AS: Adventure seeking; SD: Social desirability; E: Embitterment; TI: Trait irritability; M: Mistrust; VTA: Verbal trait aggression; PhTA: Physical trait aggression.

* *p* < 0.01,

** *p* < 0.001,

*** *p* < 0.0001.
